# Statin use and breast cancer recurrence in postmenopausal women treated with adjuvant aromatase inhibitors: a Danish population-based cohort study

**DOI:** 10.1007/s10549-020-05749-5

**Published:** 2020-06-22

**Authors:** Sixten Harborg, Uffe Heide-Jørgensen, Thomas P. Ahern, Marianne Ewertz, Deirdre Cronin-Fenton, Signe Borgquist

**Affiliations:** 1 Department of Oncology, Aarhus University Hospital/Aarhus University, Entrance C, Level 1, C118, Palle Juul-Jensens Boulevard 99, 8200 Aarhus N, Denmark; 2 Department of Clinical Epidemiology, Aarhus University, Aarhus N, Denmark; 3 Department of Surgery, University of Vermont, Burlington, USA; 4 Oncology Research Unit, Institute of Clinical Research, University of Southern Denmark, Odense C, Denmark; 5 Department of Oncology and Pathology, Lund University, Lund, Sweden

**Keywords:** Aromatase inhibitors, Cohort study, Endocrine therapy, Statins, Breast cancer

## Abstract

**Purpose:**

To examine the association between statin use and risk of breast cancer recurrence in a national Danish cohort of postmenopausal breast cancer patients receiving aromatase inhibitors (AI) in the adjuvant setting.

**Patients and methods:**

We enrolled all postmenopausal patients diagnosed with stage I–III estrogen receptor positive breast cancer during the years 2007–2017, assigned adjuvant AI treatment, and registered in both the Danish Breast Cancer Group database and the Danish Cancer Registry. We ascertained incident statin exposure (≥ 1 prescription post-diagnosis) from the Danish National Prescription Registry and modeled statins as a time-varying exposure lagged by 6 months. Follow-up began 7 months after diagnosis and continued to the first event of recurrence, death, emigration, 5 years elapsed, or 25th September 2018. We estimated incidence rates of recurrence at 5 years and used Cox regression models to compute crude and adjusted hazard ratios (HRs) with 95% confidence intervals (95% CI), comparing statin exposure with non-exposure.

**Results:**

We enrolled 14,773 eligible patients. During the 5 years of follow-up, there were 32 recurrences in 3163 person-years of follow-up among statin-exposed patients, and 612 recurrences in 45,655 person-years among unexposed patients (incidence rate per 1000 person-years: 10.12 [95% CI 6.92–14.28] and 13.40 [95% CI 12.36–14.51], respectively). In multivariable models, any statin exposure was associated with a reduced rate of 5-year breast cancer recurrence (adjusted HR 0.72 [95% CI 0.50–1.04]). Considering only lipophilic statins as exposure the results were similar (adjusted HR 0.70 [95% CI 0.48–1.02]).

**Conclusions:**

Statin use was associated with a reduced risk of breast cancer recurrence among postmenopausal patients diagnosed with early stage breast cancer who received adjuvant AI therapy.

**Electronic supplementary material:**

The online version of this article (10.1007/s10549-020-05749-5) contains supplementary material, which is available to authorized users.

## Introduction

Cholesterol-lowering medication (CLM) is frequently prescribed for prevention of cardiovascular disease [1, 2]. The most common cholesterol-lowering drugs are statins [1]. Statins are HMG-CoA reductase inhibitors, which block the rate-limiting step in the cholesterol biosynthesis [2]. Beyond this, statins may affect the incidence or severity of other diseases (e.g., cancer) by blocking the cholesterol synthesis pathway [3]. Apart from the reduction of systemic cholesterol levels through hepatic clearance, statins inhibit the mevalonate pathway in breast cancer cells, which may lower intracellular cholesterol synthesis and lead to reduced intratumoral autocrine hormone production, since cholesterol is required for the synthesis of all steroid hormones [4]. Statins may also indirectly influence tumor growth through reduced systemic levels of cholesterol and its metabolites, in particular 27-hydroxycholesterol, which acts as an estrogen receptor ligand [5–7]. Previous studies indicate an association between use of CLM and a reduced risk of breast cancer recurrence (BCR) [8]. Similarly, long-term post-diagnostic use of statins has been associated with reduced risk of contralateral breast cancer [9]. Among women with estrogen receptor positive (ER+) breast cancer, statin use seems to have a favorable impact on BCR and mortality when combined with adjuvant endocrine treatment [8, 10–12]. Lipophilic statins have been reported to have a more competent anticancer effect than hydrophilic statins [8, 13–17]. Since the publication of the earlier Danish study by Ahern et al. investigating the association between statin use and BCR, statin use has increased among Danish citizens from 23.8 defined daily doses (DDD) per 1000 inhabitants per day in 2003 to 144.6 DDD/1000 inhabitants/day in 2017 [18]. In Denmark, about 4700 women are diagnosed with breast cancer every year [19]. Given the increasing breast cancer incidence [19], any beneficial impact of statins on BCR among women treated for breast cancer could be of major value.

Aromatase inhibitors (AIs) have now been used as adjuvant therapy in postmenopausal, ER+ breast cancer patients for more than a decade. The commonly prescribed AIs, letrozole and anastrozole, both seem to increase the risk of developing hypercholesterolemia compared with tamoxifen [20, 21]. Conversely, tamoxifen treatment decreases cholesterol levels by downregulation of cholesterol synthesis [10, 22]. Hypothetically, AI-mediated estrogen suppression may thereby be less effective in women with an excessive supply of precursor hormones (e.g., as in hypercholesterolemia) since this could potentially overwhelm the anti-aromatase activity. Because AIs have been recommended as standard adjuvant treatment in Denmark—sequentially together with tamoxifen since 2007 and alone since 2009—data collected over the past decade now permit studies of recurrence risk in patients treated with AIs. This study aims to update knowledge on the association between post-diagnostic statin use and BCR in a cohort of postmenopausal patients diagnosed with early stage breast cancer, including patients diagnosed and treated in the modern era of adjuvant therapy with AIs.

We hypothesize that statin use reduces the risk of BCR among women with breast cancer, and that the protective effect is most pronounced among lipophilic statin users.

## Patients and methods

We conducted a nationwide, population-based cohort study using Danish clinical and administrative registries.

### Data sources

The Danish Breast Cancer Cooperative Group’s (DBCG) clinical database covers the entire Danish female population and includes data from 1977 with a completeness of more than 95% [23]. Patient data are reported through standardized forms by all hospital parts of the Danish health care system involved in the diagnosis, treatment, and follow-up of breast cancer [24]. The information retrieved from the DBCG registry for this study included patient age, menopausal status at diagnosis, type of primary surgery (breast-conserving surgery or mastectomy), histologic tumor type and grade, lymph node status, ER status, human epidermal growth factor receptor 2 (HER2) status, adjuvant therapy (chemotherapy, intended-to-treat endocrine therapy and radiotherapy), and clinical follow-up for recurrences. The Danish Civil Registration System (CRS) has collected data on the Danish population since 1968. The CRS is updated daily and records each person’s civil personal registration (CPR)-number, date of birth, vital and migration status [25]. The Danish National Patient Registry (DNPR) was established in 1977 and includes data on hospital-admissions, -discharge, -emergency, and -outpatient visits [26]. For each hospital encounter, one action diagnosis and up to 20 other diagnoses are recorded [27]. From the DNPR, data on comorbid diseases present at time of surgery were obtained through linkage via the CPR-number and summarized using the Charlson Comorbidity Index (CCI) [28], with breast cancer and non-melanoma skin cancer excluded. The Danish National Prescription Registry (NPR) has collected information on filled prescriptions, including anatomical therapeutic chemical (ATC) codes and date of dispensing, at hospitals and pharmacies in Denmark since 1995. However, drugs supplied directly by hospitals e.g., AIs are not captured by the registry. The completeness of NPR is high [29]. All data sources could be linked at the individual level using a unique identifier assigned to all Danish residents at birth or immigration.

### Study population

**Fig. 1:**
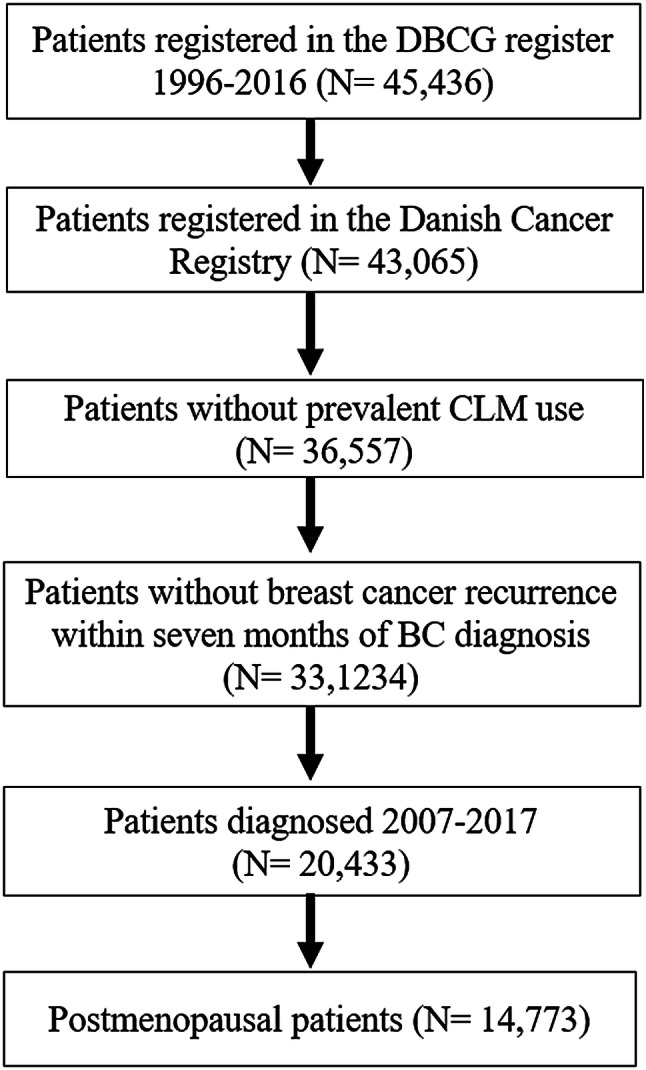
Flowchart for breast cancer patients included in the final study population. *DBCG* Danish Breast Cancer Group, *CLM* cholesterol-lowering medication, *BC* breast cancer. *Sequential treatment with Tamoxifen-Aromatase Inhibitors was recommended as standard adjuvant treatment in Denmark from 2007. In 2009, aromatase inhibitors alone became the up-front standard adjuvant treatment. **The Danish Cancer Registry is exclusively used to restrict the cohort to enable individual-level linkage with the Danish national patient registry and the Danish national prescription registry

We identified all postmenopausal women with an incident diagnosis of stage I–III ER+ breast cancer who were reported to the DBCG registry and registered in the Danish Cancer Registry between 2007 and 2017 (Fig. 1). We excluded patients who filled a dispensing of statins in the year preceding their breast cancer diagnosis. Patients were also required to be assigned to an endocrine therapy treatment protocol, per the DBCG database.

### Follow-up and statistical analysis

Follow-up began 7 months after breast cancer surgery thereby ensuring patients who had undergone adjuvant chemotherapy and had begun adjuvant endocrine treatment. Follow-up continued until any invasive BCR as recorded by DBCG, contralateral breast cancer, other malignancies, death, 5 years of follow-up, emigration or the end of available follow-up data on 25 September 2018. Patients with these events were censored at the time of the event.

For a patient to be defined as statin user in the analyses, the patient had to fill a prescription of statins. A filled prescription was assumed to expose the individual for one year of treatment. If the patient went off treatment, a new prescription was required for the patient to become exposed again. All patients redeeming a prescription of statins in the year prior to diagnosis were excluded. Statin exposure during follow-up was characterized as a time-varying variable with a 6-month lag to allow biological latency [30]. For example, a person was followed from date of surgery and assumed to be exposed 6 months after filling a statin prescription. We computed incidence rates of BCR for statin exposed and unexposed, and crude and adjusted hazard ratios using Cox regression models. Only complete cases were included in the analysis. In the adjusted model, we included the following covariates: age at diagnosis, metformin, aspirin, exposure to pre-diagnostic menopausal hormone therapy, union for international cancer control, histological grade, CCI score, type of primary surgery, adjuvant chemotherapy, and radiotherapy. Like statins, metformin and aspirin were also included as time-varying covariates. Patients filling a dispensing of metformin and/or aspirin were exposed for a year, and exposure was lagged by half a year.

### Sensitivity analyses

We conducted a series of sensitivity analyses regarding the exposure. First, we investigated our hypothesis of a more protective effect of lipophilic statins on BCR by only considering lipophilic statins as the exposure. In a further sensitivity analysis, we tried to limit misclassification of exposure in the exposed group by requiring patients to fill two prescriptions of statins in order for a subject to be classified as exposed. Finally, to examine possible differences in the association between any CLM use and breast cancer prognosis, we conducted the analyses including all types of CLM, that is, not restricted to statins.

## Results

In the cohort of 14,773 patients, median age at diagnosis was 65 years (interquartile range was 59–71 years). The median follow-up was 4.5 years and a total of 644 recurrences occurred (Table 1). Statins were prescribed to 1727 patients following breast cancer diagnosis. Most patients underwent breast-conserving surgery (68.9%) while the rest had undergone mastectomy (31.1%). At baseline, the CCI score was low for most patients. However, those who initiated statins during follow-up had more comorbidity and were older compared with non-users. Beyond this, incident statin users were more frequently assigned breast cancer conserving surgery and adjuvant radiotherapy. In addition, statin users were more likely to be treated with metformin (*N* = 306 [17.7%]) and aspirin (*N* = 566 [32.8%]) compared with non-users (*N* = 327 [2.4%]), (*N* = 1355 [10.1%]), respectively.

**Table 1:** Patient and disease characteristics of postmenopausal women diagnosed with early stage breast cancer in Denmark from 2007–2017

	Statin ever users, *N* (%)	Statin never users, *N* (%)
Total	1727	13,046
Age at diagnosis (years)		
30–39	0 (0.0)	5 (0.0)
40–49	6 (0.3)	174 (1.3)
50–59	423 (24.5)	4007 (29.9)
60–69	875 (50.7	5510 (41.1)
70–79	356 (20.6)	2614 (19.5)
80+	67 (3.9)	1094 (8.2)
UICC stage		
I	656 (38.0)	5247 (39.1)
II	822 (47.6)	6093 (45.4)
III	205 (11.9)	1606 (12.0)
Missing	44	460
Type of primary surgery		
Mastectomy	494 (28.6)	4216 (31.4)
Breast-conserving surgery/lumpectomy	1233 (71.4)	9190 (68.6)
Adjuvant chemotherapy	405 (23.5)	3976 (29.7)
Adjuvant radiotherapy	672 (38.9	4036 (30.1)
Metformin	306 (17.7)	327 (2.4)
Aspirin	566 (32.8)	1355 (10.1)
Hormone replacement therapy	1021 (59.1)	7698 (57.4)
Charlson Comorbidity Index score		
None (score 0)	1247 (72.2)	10,325 (77.0)
Mild (score 1–2)	389 (22.5	2455 (18.3)
Severe (score 3+)	91 (5.3)	626 (4.7)
Histological grade		
Grade 1	434 (25.1)	3173 (23.7)
Grade 2	898 (52.0)	7101 (53.0)
Grade 3	283 (16.4)	2147 (16.0)
Missing	112	985
Histological type		
Ductal	1452 (84.1)	10,835 (80.8)
Lobular	172 (10.0)	1701 (12.7)
Other/misisng	103	870
HER2		
Normal	1427 (82.6)	11,265 (84.0)
Overexpressed	191 (11.1)	1521 (11.3)
Missing	109	620

*UICC* Union for International Cancer Control, *CCI* Charlson Comorbidity Index, *HER2* human epidermal growth factor receptor 2

Follow-up ended at the 25th of September 2018

Non-lobular and non-ductal breast cancers are not histologically graded

Table 2 displays the estimated hazard ratios in relation to statin use during the 5 years of follow-up. A total of 32 recurrences occurred during 3163 person-years of follow-up among women in the study population exposed to statins and 612 recurrences in 45,655 person-years in the unexposed group (incidence rate per 1000 person-years: 10.12 [95% CI 6.92–14.28] and 13.40 [95% CI 12.36–14.51], respectively). We observed a reduced risk of BCR associated with incident statin exposure (adjusted HR 0.72 [95% CI 0.50–1.04]). The sensitivity analyses restricted to lipophilic statin use (Table 2) yielded similar findings (adjusted HR 0.70 [95% CI 0.48–1.02]). The association held up among patients that filled two prescriptions of statins (adjusted HR 0.75 [95% CI 0.51–1.11]), (Table 2). The association was not observed in analyses restricted to hydrophilic statin use (adjusted HR 0.73 [95% CI 0.18–2.91]). Analyses not restricted to statins but including all types of CLM did not attenuate the association (adjusted HR 0.75 [95% CI 0.53–1.07]) (Table 2).

**Table 2:** Number of recurrences and recurrence rates per 1000 person-years in relation to statin use after breast cancer surgery in postmenopausal women on AIs diagnosed in Denmark from 2007–2017

Exposure	Person-years	Recurrences	Incidence rate per 1000 person-years (95% CI)	Hazard ratio (95% CI)	Adjusted hazard ratio (95% CI)
Not exposed to CLM	45,596	610	13.38 (12.34–14.48)		
CLM exposure	3231	34	10.52 (7.29–14.70)	0.75 (0.53–1.07)	0.75 (0.53–1.07)
Not exposed to statins	45,655	612	13.40 (12.36–14.51)		
Statin exposure	3163	32	10.12 (6.92–14.28)	0.72 (0.50–1.03)	0.72 (0.50–1.04)
Not exposed to lipophilic statins	45,787	614	13.41 (12.37–14.51)		
Lipophilic statin exposure	3041	30	9.87 (6.66–14.08)	0.70 (0.49–1.02)	0.70 (0.48–1.02)
Not exposed to simvastatins	46,506	619	13.31 (12.28–14.40)		
Simvastatin exposure	2231	25	10.77 (6.97–15.90)	0.78 (0.52–1.16)	0.73 (0.48–1.10)
Not exposed to atorvastatins	48,000	639	13.31 (12.30–14.39)		
Atorvastatin exposure	827	5	6.05 (1.96–14.11)	0.44 (0.18–1.05)	0.54 (0.22–1.31)

*CI* confidence interval, *CLM* cholesterol-lowering medication

Adjusted for; age at diagnosis; union for international cancer control; histological grade; adjuvant chemotherapy; type of primary surgery; radiotherapy; hormone therapy; metformin; aspirin

## Discussion

The results of this study show an association between post-diagnostic incident statin use and a reduction in risk of recurrence among patients diagnosed with early breast cancer and treated with aromatase inhibitors. Beyond this, the study also displays a reduction in risk of recurrence among these patients when exclusively exposed to lipophilic statins in accordance with earlier studies [8, 13]. This study, performed in a modern cohort of AI-treated breast cancer patients, supports the findings from earlier studies, based on predominantly tamoxifen-treated populations [8, 12, 13, 31–35].

In cancer cells, statins are associated with cell cycle disruption [36]. Statins exert pleiotropic effects through their ability to decrease levels of farnesyl pyrophosphate and geranylgeranyl pyrophosphate, thereby decreasing cellular signaling of G proteins [37], and restrain proliferation and survival of cancer cells [38]. Lipophilic statins diffuse across the plasma membrane in extrahepatic cells, thus disrupting cholesterol synthesis, whereas hydrophilic statins are largely confined to the liver [39]. This suggests that lipophilic statins can also affect breast cancer cells directly, whereas hydrophilic statins, solemnly affect breast cancer cells indirectly via alterations in systemic cholesterol levels [40]. The majority of statin users in this study were exposed to simvastatin and a large amount to atorvastatin, both regarded as being lipophilic statins. This is in accordance with our hypothesis, that lipophilic statins have a more competent anticancer effect. Nonetheless, it makes the generalizability of the association between statins in general and a reduced risk of BCR limited to the most commonly prescribed statins.

Considering the association of higher recurrence rates amongst overweight and obese women diagnosed with breast cancer [41, 42], and the associations between statins and overweight [43], it would have been of importance to account for body mass index as a confounder. Due to limited data access, this was unfortunately not possible. As no adjustment for BMI was made in the analyses of this study it may have impacted the results and made them more imprecise compared to results adjusted for BMI. Another possible limitation to this study may be adherence to endocrine therapy. Non-adherence to adjuvant endocrine therapy is a clinically relevant concern, and several studies have observed the negative influence on clinical outcome [44]. The most critical factor to prevent discontinuation of endocrine therapy is reported to be patient contact [45]. In addition, adherence to statin therapy has been reported to be low in cardiovascular studies [46]. Moreover, patient contact has been highlighted as an important factor in adherence to statins [47]. Yet, discontinuation of statin therapy is often not permanent, and as many as 60% of the patients discontinuing statin therapy returns to their medication plan within 2 years [48]. Nonetheless, patients within this cohort, are closely monitored throughout their first 5 years of endocrine treatment after breast cancer diagnosis [49]. However, in this study it was not possible to control for accuracy of classification of adjuvant endocrine treatment—nor for adherence to therapy—as an intention-to-treat approach was used. Yet, studies in DBCG report high compliance in treatment patterns to the national guidelines [23]. Therefore we do not expect our results to be due to change in endocrine treatment, as this patient group is engaged in one type of endocrine treatment, and will change only if treatment is contraindicated [50].

Systemic cholesterol levels are associated with both risk [51] and prognosis [52] of breast cancer. As hypercholesterolemia is one of the main indications for a statin prescription [53], it could possibly induce confounding by indication to influence our results.

Another potential bias might be that higher cholesterol levels are a function of particularly robust response to AI therapy; however, earlier studies by us employed a marginal structural modeling to account for e.g., time-varying cholesterol levels, and the clinically beneficial associations between statin use and BCR held up [10].

The decreased risk of recurrence associated with statin use in our study may also be due to healthy user bias. However, in Denmark, statins are only available via prescription and a Danish study concluded that statin users are representative of the general Danish population [54], an inverse association is therefore unlikely. Unfortunately, no information on mammography screening history was available; therefore, statin users might induce surveillance bias if incident statin users were more prone to undergo breast cancer surveillance. This could also possibly induce lead-time bias by increasing the likelihood of breast cancer diagnosis at an earlier stage. However, our analyses were adjusted for disease stage.

In this study anyone redeeming a statin prescription in the year prior to diagnosis was excluded, a time-varying approach in modeling the analyses was used to eliminate immortal time bias and drug exposure was lagged by one year to avoid reverse causation [55]. Finally, we do not expect considerable residual confounding of our adjusted estimates since the associations were not affected by adjustment for factors associated with recurrence. Other factors than the ones adjusted for that should be strongly associated with both statin use and BCR is considered unlikely to exist.

Our study provide evidence for a reduced risk of recurrence associated with statin use among postmenopausal women with an early stage breast cancer who are treated with AIs. Taken together with previous studies [8], this study provides consistent consecutive association between statin use and a reduction in BCR over time in a high-quality database. These findings suggest that AI-treated, early stage breast cancer patients might benefit further from addition of a lipophilic statin to their adjuvant therapy regimen. This question warrants investigation in a properly randomized study to decide statins definite role in breast cancer. Prospective research should aim to investigate how statins can decrease the risk of BCR and cardiovascular diseases as a repercussion of breast cancer treatment with AIs.

## Conclusion

In conclusion, our study confirms the previously reported recurrence benefit of statins in postmenopausal early breast cancer patients, studied for the first time in the modern AI treatment era. The evidence from this study supports further investigation of adjuvant statin therapy in a randomized clinical trial of breast cancer patients.

## Electronic supplementary material

Below is the link to the electronic supplementary material.

Supplementary file1 (DOCX 23 kb)

## Data Availability

The data that support the findings of this study are available from *Danish Breast Cancer Cooperative Group*, but restrictions apply to the availability of these data, which were used under license for the current study, and so are not publicly available. Data are however available from the authors upon reasonable request and with permission of *Danish Breast Cancer Cooperative Group*.

## Abbreviations

AI: Aromatase inhibitors
HR: Hazard ratio
CI: Confidence interval
CLM: Cholesterol-lowering medication
BCR: Breast cancer recurrence
ER+: Estrogen receptor positive
DDD: Defined daily doses
DBCG: Danish Breast Cancer Cooperative Group
HER2: Human epidermal growth factor receptor 2
CRS: The Danish civil registration system
CPR: Civil personal registration
DNPR: Danish national patient registry
CCI: Charlson Comorbidity Index
NPR: The Danish national prescription registry
ATC: Anatomical therapeutic chemical
BMI: Body mass index
