# Supplementary material for: Statin use and breast cancer recurrence in postmenopausal women treated with adjuvant aromatase inhibitors: a Danish population-based cohort study
Source: Breast Cancer Res Treat. 2020 Jun 22;183(1):153–60. doi: 10.1007/s10549-020-05749-5 (PMC7376511; doi:10.1007/s10549-020-05749-5)
Supplement: Supplementary file 1 — Supplementary file1 (DOCX 23 kb) [file 10549_2020_5749_MOESM1_ESM.docx]

***APPENDIX***

**Supplemental table 1** Characteristics of statin medications prescribed to stage I, II, or III breast cancer patients diagnosed in Denmark from 2007 to 2017.

| **Drug name** | **ATC code** | **N (%)** |
| --- | --- | --- |
| Simvastatin | C10AA01 | 9,646 (62.9) |
| Lovastatin | C10AA02 | <5 (0.0) |
| Pravastatin | C10AA03 | 78 (0.5) |
| Atorvastatin | C10AA05 | 4,197 (27.3) |
| Rosuvastatin | C10AA07 | 865 (5.6) |
| Gemfibrozil | C10AB04 | 42 (0.3) |
| Colestyramin | C10AC01 | 113 (0.7) |
| Nicotinic acid and derivatives | C10AD52 | 34 (0.2) |
| Ezetimibe | C10AX09 | 372 (2.4) |
